# Evaluation of Shoulder Mobility After Breast Reconstruction With a Lipofilled Latissimus Dorsi Mini‐Flap: A Prospective Cohort Study

**DOI:** 10.1155/tbj/5107548

**Published:** 2026-03-08

**Authors:** Bruno Carvalho Carelli, Fabio Bagnoli, Eduardo de Melo Carvalho Rocha, José Francisco Rinaldi, Vilmar Marques de Oliveira

**Affiliations:** ^1^ Faculty of Medical Sciences of the Santa Casa de São Paulo, São Paulo, Brazil

**Keywords:** breast reconstruction, latissimus dorsi, lipofilling, mini-flap, shoulder function

## Abstract

**Background:**

The lipofilled latissimus dorsi mini‐flap (LDMF‐L) broadens autologous breast‐reconstruction options, yet its functional impact on the shoulder remains uncertain.

**Objective:**

To evaluate shoulder strength, range of motion (ROM) and patient‐reported upper‐limb function QuickDash 90 days after breast reconstruction with the LDMF‐L.

**Methods:**

Prospective cohort of 20 patients operated on between November 2022 and November 2024. Inclusion: Breast cancer requiring immediate or delayed reconstruction with LDMF‐L; exclusion: Implant use or major pre‐existing limitation. Strength (Oxford scale), ROM (goniometry) and QuickDASH score were assessed preoperatively and at 90 days. Wilcoxon, Student′s *t*‐test, Mann–Whitney and McNemar tests were used appropriately (*α* = 0.05).

**Results:**

Mean age 54 ± 11.8 years; immediate/delayed reconstruction = 50/50%. Strength remained unchanged in 85% (*p* = 1.000). Active flexion and abduction showed significant reductions (*p* = 0.016 and 0.045), with no difference in rotations. QuickDASH increased from 8 ± 16 to 19 ± 24 (*p* = 0.008); nevertheless, 80% stayed within minimal/mild disability.

**Conclusions:**

The LDMF‐L preserves strength and produces only mild early ROM decreases with limited functional impact, supporting its functional safety as an implant‐free autologous option.

## 1. Introduction

Breast cancer remains the most common malignancy and leading cause of cancer‐related death among Brazilian women, accounting for ∼31% of female tumours [1]. Advances in screening and adjuvant therapy have raised 5‐year survival to 75%, making posttreatment quality of life a critical endpoint [2]. Autologous breast reconstruction with the latissimus dorsi (LD) flap is versatile and safe, yet its limited volume often mandates implants, which carry their own complications [3].

The extended LD flap provides extra volume but increases donor‐site morbidity and seroma rates [4–7]. The latissimus dorsi mini‐flap (LDMF) introduced by Raja et al. [8] preserves most of the muscle and avoids large dorsal incisions; when combined with lipofilling (LDMF‐L), it seeks to minimise donor‐site morbidity while supplying adequate breast volume without implants [9, 10].

Despite favourable aesthetic results, the functional consequences on the shoulder remain unclear, given the LD role in flexion, abduction and rotation [3]. Previous studies addressed conventional or extended LD flaps, but few examined the mini‐flap technique [4, 11]. We therefore prospectively quantified strength, ROM and self‐reported disability in the early postoperative period.

## 2. Methods

### 2.1. Study Design and Participants

This prospective cohort was approved by the Institutional Review Board of the Santa Casa de São Paulo School of Medicine. Twenty women undergoing immediate or delayed reconstruction with the LDMF‐L between November 2022 and November 2024 provided written informed consent. Exclusion criteria were concomitant implant placement and significant pre‐existing shoulder impairment.

### 2.2. Surgical Procedure

Patients remained in the supine position throughout, eliminating intraoperative repositioning. Tumescent solution (500 mL saline + adrenaline 1:1 000 000) was infiltrated, followed by syringe liposuction of donor areas (abdomen, thighs and flanks). The harvested fat was filtered and decanted without centrifugation. Lipofilling is performed before flap dissection.

The flap was dissected by initially identifying the serratus anterior medially, proceeding laterally along this plane. The partial LD detachment beneath the skin paddle preserved maximal muscle volume. The thoracodorsal pedicle was visualised, maintaining the descending branch within the flap. A subcutaneous tunnel delivered the flap to the breast; additional lipofilling achieved contour and volume. Drains were placed in the breast and donor site.

Patients wore a surgical bra for 30 days and were instructed to avoid arm elevation >°90° during that period. Average hospital stay was 1‐2 days.

### 2.3. Functional Assessments

• Strength: Strength was assessed using the Oxford scale (0–5).
• ROM: ROM was measured by goniometry for active flexion, abduction, external and internal rotation.
• Patient‐reported function: This was evaluated using QuickDASH questionnaire (11 items, 0%–100%).

Assessments were performed preoperatively (T0) and at 90 days (T1) by a specialised physiatry team.

### 2.4. Statistical Analysis

Analyses were performed using SPSS Version 25 (IBM Corp., Armonk, NY). Data are presented as mean, median, standard deviation and percentages. Normality of quantitative variables was verified using the Shapiro–Wilk test. Paired quantitative variables with normal distribution were analysed using the paired *Student’s t*‐test, whereas non‐normally distributed paired variables were analysed using the *Wilcoxon signed-rank test*. Comparisons between independent groups were performed using the *Mann–Whitney U test*. Categorical paired variables were assessed using McNemar’s test. Statistical significance was set at *p* < 0.05.

## 3. Results

Of 25 initial cases, five were excluded (three lost to follow‐up, one complication and one death). Twenty patients were analysed; half underwent immediate reconstruction (Table 1).

**TABLE 1:** Sample characteristics.

Characteristic	Total (*n* = 20) (%)	Delayed reconstruction (*n* = 10) (%)	Immediate reconstruction (*n* = 10) (%)
Age (mean, years)	54	56.2	51.7

*BMI*			
Normal Overweight Obesity	11 (55) 7 (35) 2 (10)	7 (70) 1 (10) 2 (20)	4 (40) 6 (60) 0 (0)

*Smoking*			
Yes No	2 (10) 18 (90)	2 (20) 8 (80)	0 (0) 10 (100)

*Radiotherapy*			
Yes No	20 (100) 0 (0)	10 (100) 0 (0)	10 (100) 0 (0)

*Nodal dissection*			
Axillary dissection Sentinel lymph node	12 (60) 8 (40)	8 (80) 2 (20)	4 (40) 6 (60)

*Note:* Classification: underweight (< 18.5), normal (18.5–24.9), overweight (25–29.9) and obesity (≥ 30). Age expressed as mean complete years. Other variables are presented as absolute frequency with the corresponding percentage in parentheses.

Abbreviation: BMI = Body Mass Index.

### 3.1. Muscle Strength

Strength did not change significantly from baseline, with 85% of patients retaining their Oxford score. Two patients (10%) decreased to Grade 4, retaining full range against gravity with partial resistance (Table 2).

**TABLE 2:** Preoperative and 90‐day postoperative strength evaluation using the Oxford scale.

			Strength T90		Total
			4	5	
Strength T0	4	*N*	2	1	3
		%	10%	5%	15%
	5	*N*	2	15	17
		%	10%	75%	85%

Total		*N*	4	16	20
		%	20%	80%	100%

*Note:* Test McNemar (*p* = 1000). *N* = absolute frequency, *T*0 = preoperative and T90 = 90‐day postoperative.

### 3.2. Range of Motion

Physiatric evaluation showed significant reductions in active flexion (*p* = 0.016) and abduction (*p* = 0.045). External and internal rotations were unaffected. Using ≤°15° loss as clinically irrelevant, 65% of patients fell below this threshold; 95% had minimal changes in rotations (Tables 3, 4, 5).

**TABLE 3:** Shoulder ROM: quantitative difference between the 90‐day postoperative and preoperative measurements.

		*N*	Mean rank	Sum of ranks	*p* value
Active flexion	Negative ranks	8	6.38	51	0.016
	Positive ranks	2	2	4	
	Ties	10	—	—	
	Total	20	—	—	

Active abduction	Negative ranks	9	7.17	64.50	0.045
	Positive ranks	3	4.50	13.50	
	Ties	8	—	—	
	Total	20	—	—	

Active internal rotation	Negative ranks	3	6	18	0.155
	Positive ranks	8	6	48	
	Ties	9	—	—	
	Total	20	—	—	

*Note:* Wilcoxon test. *N* = absolute frequency.

**TABLE 4:** Shoulder ROM for external rotation: quantitative difference between the 90‐day postoperative and preoperative measurements.

	Mean	*N*	*p* value
Active external rotation T0	62.75	20	0.762

Active external rotation T90	61.75	20	

*Note:* Student′s *t*‐test for paired samples. *N* = absolute frequency; *T*0 = preoperative; T90 = postoperative 90 days.

**TABLE 5:** Qualitative analysis of shoulder range of motion measured with a goniometer.

Variable	% worsened (%)	95% CI lower limit (%)	95% CI upper limit (%)
Active flexion	35	14.1	55.9
Active abduction	35	14.1	55.9
External rotation	5	0.0	16.9
Internal rotation	5	0.0	16.9

*Note:* 95% CI = 95% confidence interval; “%” = percentage; lower/upper limits correspond to the confidence bounds.

### 3.3. QuickDASH

**FIGURE 1:**
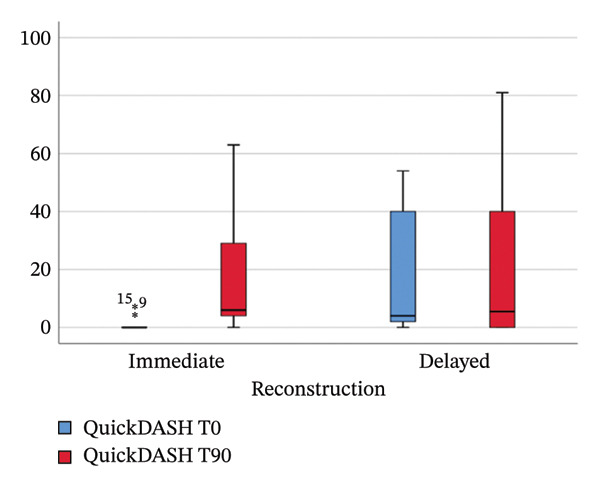
Median QuickDASH scores preoperatively (T0) and at 90 days (T90) in immediate and delayed reconstruction groups. *T*0 = preoperative; T90 = 90‐day postoperative.

QuickDASH increased significantly at 90 days (*p* = 0.008). Although 55% reported worsening, 80% remained within minimal/mild disability (0–40) (Figure 1).

Delayed reconstruction patients—who had previously undergone mastectomy and axillary surgery without LD reconstruction—presented significantly higher baseline QuickDASH scores compared with the patients scheduled for immediate reconstruction (*p* = 0.011), indicating worse upper‐limb functional status before reconstruction in patients previously treated with oncologic surgery alone (Table 6). Despite this worse baseline condition, 60% of these patients maintained or improved their functional status at the 90‐day postoperative evaluation (Figure 1). *Immediate reconstruction* patients showed a significant score increase (*p* = 0.012) with 70% worsening; however, 80% still had mild disability.

**TABLE 6:** Preoperative QuickDASH score in immediate versus delayed reconstruction.

Reconstruction		*N*	Mean rank	Sum of ranks	*p* value
QuickDASH t0	Immediate	10	7.35	73.5	0.011
	Delayed	10	13.65	136.5	
	Total	20			

*Note:* Teste de Mann–Whitney test. *t*0 = preoperative; *N* = absolute frequency.

### 3.4. Axillary Surgery

To assess the independent impact of axillary surgery, we performed a comparative analysis between patients submitted to sentinel lymph node biopsy and those who underwent axillary dissection using the Mann–Whitney *U* test.

At baseline (preoperative evaluation), no statistically significant differences were found between the two groups regarding shoulder range of motion, muscle strength, or QuickDASH scores, indicating comparable functional status regardless of axillary approach.

At the 90‐day postoperative evaluation, no statistically significant differences were observed between the sentinel and axillary dissection groups in QuickDASH scores, physiatric assessment of muscle strength or range of motion. These findings suggest that, in our cohort, the type of axillary surgery did not independently influence early postoperative shoulder functional outcomes (Table 7).

**TABLE 7:** Pre‐ to 90‐day postoperative functional variation in LDMF patients: sentinel lymph node biopsy versus axillary lymph node dissection.

Axilla		N	Mean rank	Sum of ranks	*p* value
Q‐DASH	Axillary lymph node dissection	12	11.33	136	0.409
	Sentinel lymph node biopsy	8	9.25	74	
	Total	20	—	—	

Flexion	Axillary lymph node dissection	12	10.54	126.50	0.969
	Sentinel lymph node biopsy	8	10.44	83.50	
	Total	20	—	—	

Abduction	Axillary lymph node dissection	12	10.04	120.50	0.667
	Sentinel lymph node biopsy	8	11.19	89.50	
	Total	20	—	—	

*Note:* Mann–Whitney test.

## 4. Discussion

Our goal was to evaluate shoulder strength and mobility after breast reconstruction with the LDMF‐L. Although numerous studies have examined LD‐related functional changes, few focus on the mini‐flap [4, 11].

Early (3 months) assessment was chosen to determine whether the muscle‐sparing nature of the mini‐flap translates into lower morbidity. Steffenssen et al. [6] systematically reviewed shoulder morbidity after LD reconstruction, noting near‐normal function by 6–12 months with the greatest deficits during the first 3 months; they advocated studies using DASH and expert‐performed goniometry—both applied here.

Our findings confirm that LDMF‐L preserves strength and causes only mild ROM reduction at 90 days, mirroring meta‐analyses of conventional LD reporting transient flexion/abduction deficits [6]. Maintaining strength in 85% suggests less muscle sacrifice with the mini‐flap [12].

The modest QuickDASH increase, while statistically significant, remained clinically mild. Immediate reconstructions, owing to the absence of prior surgery, exhibited worse postoperative outcomes. Delayed reconstructions, meanwhile, began with higher QuickDASH scores but showed no significant deterioration.

The functional impact of breast and axillary surgery in the absence of LD reconstruction can be inferred from the significantly higher preoperative QuickDASH scores observed in the delayed reconstruction group. These patients had already undergone mastectomy and axillary surgery without immediate reconstruction and exhibited worse baseline upper‐limb function compared with the patients submitted to immediate reconstruction. This finding suggests that mastectomy and axillary surgery alone are associated with measurable functional impairment of the shoulder.

This observation is consistent with previous reports in the literature. Oliveira et al. demonstrated that patients submitted to mastectomy and axillary dissection show significant postoperative shoulder dysfunction even in the absence of flap reconstruction. Therefore, part of the functional limitation observed in breast cancer patients should be attributed to the oncologic procedure itself rather than exclusively to the reconstructive technique [13].

Duymaz et al. [4] studied mini‐flap reconstruction after wide local excision and found significant QuickDASH differences when combined with axillary dissection; 69.5% showed no mobility decline at 21–34 months. Our larger early deficits may reflect the shorter follow‐up. Zhou et al. [11] compared mini‐flap reconstruction after breast‐conserving surgery and found no functional differences at 1 year.

Longer follow‐up is warranted to confirm functional recovery beyond 1 year, and structured rehabilitation protocols may further optimise outcomes. This study has limitations. The small sample size may have reduced statistical power and limited the generalisability of our findings. As a single‐arm prospective cohort, direct comparison with other reconstruction techniques was not possible, and full blinding of evaluators was inherently unfeasible because of the visible anatomical features of reconstructive surgery.

Because each patient served as her own control through pre‐ and postoperative assessments, the study design allowed a precise appraisal of the functional impact of the LDMF. Despite these constraints, the prospective methodology and the use of standardised physiatric evaluations strengthen the reliability of the functional outcomes reported.

## 5. Conclusions

Breast reconstruction with the LDMF‐L is functionally safe. At 90 days, shoulder strength is preserved, and ROM reductions are limited to flexion/abduction with minimal functional repercussion in most patients. The technique offers an implant‐free autologous alternative with low donor‐site morbidity.

## Funding

No funding was received for this manuscript.

## Conflicts of Interest

The authors declare no conflicts of interest.

## Data Availability

The data that support the findings of this study are available on request from the corresponding author. The data are not publicly available due to privacy or ethical restrictions.

## References

[1] Brazilian National Cancer Institute (Inca) , Estimate 2023: Cancer Incidence in Brazil, 2022.

[2] Ferreira A. S. S. , Cintra J. R. D. , Fayer V. A. et al., Breast Cancer Survival and the Health System in Brazil: An Analysis of Public and Private Healthcare, Frontiers in Oncology. (2023) 13, 10.3389/fonc.2023.927748. PMC10248159 37305573

[3] Spear L. and Hess C. L. , A Review of the Biomechanical and Functional Changes in the Shoulder Following Transfer of the Latissimus Dorsi Muscle, Plastic and Reconstructive Surgery. (2005) 115, no. 7, 2070–2073, 10.1097/01.PRS.0000163329.96736.6A, 2-s2.0-20044370646. 15923857

[4] Duymaz T. , İyigün Z. E. , İlgün A. S. et al., The Effect of Mini-Latissimus Dorsi Flap (MLDF) Reconstruction on Shoulder Function in Breast Cancer Patients, European journal of breast health. (2019) 15, no. 3, 158–162, 10.5152/ejbh.2019.4727. 31312791 PMC6619775

[5] Mushin O. P. and Langstein H. N. , Indications for implant-enhanced Latissimus Dorsi Reconstruction, Clinics in Plastic Surgery. (2018) 45, 113–121. 10.1016/j.cps.2017.08.006 29080662

[6] Steffenssen M. C. W. , Kristiansen A. L. H. , and Damsgaard T. E. , Functional Shoulder Impairment After Latissimus Dorsi Breast Reconstruction: A Systematic Review and meta-analysis, Annals of Plastic Surgery. (2019) 82, no. 1, 116–127, 10.1097/SAP.0000000000001691, 2-s2.0-85058601778. 30516558

[7] Brondi R. S. , de Oliveira V. M. , Bagnoli F. , Mateus E. F. , and Rinaldi J. F. , Autologous Latissimus Dorsi Flap with Immediate Fat Grafting, Annals of Plastic Surgery. (2019) 82, 321–327. 10.1097/SAP.0000000000001764 30570566

[8] Raja M. A. , Straker V. F. , and Rainsbury R. M. , Extending the Role of breast-conserving Surgery by Immediate Volume Replacement, British Journal of Surgery. (1997) 84, no. 1, 101–105, 10.1002/bjs.1800840138, 2-s2.0-0031016553. 9043470

[9] Escandón J. M. , Escandón L. , Ahmed A. et al., Breast Reconstruction Using the Latissimus Dorsi Flap and Immediate Fat Transfer (LIFT): A Systematic Review and meta-analysis, Journal of Plastic, Reconstructive & Aesthetic Surgery. (2022) 75, no. 11, 2470–2481, 10.1016/j.bjps.2022.08.025. 36241504

[10] Schwabegger A. H. , Harpf C. , and Rainer C. , Muscle-Sparing Latissimus Dorsi Flap, Plastic and Reconstructive Surgery. (2003) 112, 804–811, 10.1097/01.PRS.0000049448.56511.23, 2-s2.0-0037375745. 12618599

[11] Zhou L. , Wang Y. , Cai R. et al., Descending-Branch Pedicled Latissimus Dorsi mini-flap for Partial Mastectomy Defects: Functional and Aesthetic Outcomes, Journal of Surgical Oncology. (2019) 120, no. 3, 518–526, 10.1002/jso.25524, 2-s2.0-85070201175. 31168844

[12] Garusi C. , Manconi A. , Lanni G. et al., Shoulder Function After Breast Reconstruction with the Latissimus Dorsi Flap: A Prospective Cohort Study Combining DASH Score and Objective Evaluation, The Breast. (2016) 27, 78–86, 10.1016/j.breast.2016.02.017, 2-s2.0-84962030024. 27054752

[13] Oliveira R. R. , Nascimento S. L. , Derchain S. F. M. , and Sarian L. O. , Immediate Breast Reconstruction with a Latissimus Dorsi Flap Has No Detrimental Effects on Shoulder Motion or Postsurgical Complications up to One Year After Surgery, Plastic and Reconstructive Surgery. (2013) 131, 673e–680e, 10.1097/PRS.0b013e31828659de, 2-s2.0-84877765354. 23629106

